# Dependence of the Distribution and Structure of the White Mulberry (*Morus alba*) Population in Wrocław on the Intensity of Anthropopressure and Thermal Conditions

**DOI:** 10.3390/ijerph19020838

**Published:** 2022-01-12

**Authors:** Katarzyna Blitek, Daniel Pruchniewicz, Przemysław Bąbelewski, Marta Czaplicka-Pędzich, Marcin Kubus

**Affiliations:** 1 Department of Horticulture, Faculty of Life Sciences and Technology, Wrocław University of Environmental and Life Sciences, Pl. Grunwaldzki 24a, 50-363 Wrocław, Poland; przemyslaw.babelewski@upwr.edu.pl (P.B.); marta.czaplicka-pedzich@upwr.edu.pl (M.C.-P.); 2 Department of Botany and Plant Ecology, Faculty of Life Sciences and Technology, Wrocław University of Environmental and Life Science. Pl. Grunwaldzki 24a, 50-363 Wrocław, Poland; daniel.pruchniewicz@upwr.edu.pl; 3 Department of Landscape Architecture, West Pomeranian University of Technology, Ul. Papieża Pawła VI Str 3a, 71-459 Szczecin, Poland; marcin.kubus@zut.edu.pl

**Keywords:** white mulberry, population, environment, Wrocław, ecology

## Abstract

The selection of species which show the highest possible tolerance to negative habitat conditions, also among plants of foreign origin, is a pressing issue. One of the species we would like to recommend for planting in urban areas is the white mulberry species (*Morus alba)* due to both its outstanding adaptability and its ecosystem services. There are no reliable studies on the distribution of this species in urbanized areas in Poland, nor sufficient analyses of the methods of its renewal, both deliberate and spontaneous spread through self-seeding. Collecting data on the population of an alien species within individual regions and forecasting potential changes in the population’s size and structure, as well as its possible impacts on other organisms, is one of the basic measures to reduce biological invasions, which is one of the six priority objectives of the European Biodiversity Strategy and an element of the Strategy on Invasive Alien Species. The aim of this study was to determine the size and structure of the white mulberry population in the city of Wrocław and to analyse the relationship between this structure and intensity of anthropopressure and thermal conditions.

## 1. Introduction

Numerous studies of the population structure and methods of natural and artificial spread of alien taxa of forest trees have already been conducted in Poland, but the number of such studies focusing on urbanized areas has so far been insufficient [1,2]. It is known that trees improve the quality of urban environment-they purify the air by absorbing harmful gases, dust, and excess CO_2_. Moreover, they produce oxygen, promote the renewal of water resources and reduce rainwater runoff, thus preventing flooding. They also regulate local microclimate, e.g., by lowering the temperature through shading, and provide protection against the wind. Trees are an inseparable element of a city landscape, its historical and cultural identity, having a positive impact on both physical and mental health of the inhabitants, as well as on the value of the local real estate. 

However, there are also risks associated with their presence in urban spaces [3,4,5,6,7]. Unfortunately, the human-designed urban environment is extremely unfavorable for trees. Anthropogenic stress factors, such as air and soil pollution, in addition to the inadequate structure and pH of the substrate, lack of symbiotic microorganisms or mechanical damage limit tree growth and thus the development of plants in certain areas of city agglomeration, e.g., those located near busy arterial roads, or industrial and densely built-up areas. Increasingly frequent periods of drought and heat waves are associated not only with climate warming, but also with the phenomenon of the so-called Urban Heat Island [8,9]. Moreover, over 70% of rainwater in cities is discharged into the sewage system, and due to the predominance of paved surfaces, retention is severely curtailed, which further reduces the availability of moisture that could be used by plants. Weakened trees are more prone to disease and pests, and in extreme cases, they may die [10,11,12]. The selection of species which show the highest possible tolerance to negative habitat conditions is thus a pressing issue. According to numerous studies, environmental variables affect the health status and the possibility of natural and artificial renewal in the population of specific taxa [3,13,14,15,16]. 

Currently, the selection of native species suitable for planting in urban areas is becoming more and more difficult because many of them are not able to survive in specific urban conditions. Studies on the preservation and behaviour of native tree species in urban space, especially regarding the selection of candidates for planting in agglomerations, should be continued. However, it becomes necessary to search for tree species and varieties that do not occur naturally in a given country but are capable of withstanding strong anthropopressure. One of the species that is worth recommending for use in urban areas is the white mulberry (*Morus alba*), due to both its outstanding adaptability and its ecosystem services. This tree species grows up to approximately 15 m, with a picturesque habit, a wide crown and highly varied foliage, also suitable for cultivation in the form of rows or hedges [17]. Although it is classified as a thermophilic plant, it can withstand temperature drops to −30 °C, and its stems quickly regenerate after freezing. Moreover, it starts growing late–in Poland, the beginning of bud development occurs in April–May, and so the buds are usually not damaged by spring frosts [18]. The species is also extremely tolerant of soil conditions–it tolerates both drought and transient wetlands, although response to water stress differs depending on the cultivar. White mulberry is characterized by considerable resistance to air pollution, as well as soil salinity, increased content of heavy metals, sulfur, and calcium compounds in the soil. It is used in reclamation projects and planted along busy arterial roads [19,20,21,22,23,24,25,26,27,28,29,30,31,32,33]. It has a long lifespan–growing in the wild its age often exceeds 500 years, and in Poland there are single specimens estimated to be over 250 years old, including the white mulberry tree growing in Sulechów, which has been recognized as a natural monument and has the largest trunk circumference in Europe (412 cm) [https://www.sulechow.pl/pomniki-przyrody.html, accessed on 9 March 2020]. Due to its high annual growth of biomass, white mulberry has a great capacity to store CO_2_ in its tissues, hence it is especially valuable for urban areas [34,35]. 

For centuries, its leaves have been used as a herbal remedy (raw material); it also provides tasty, valued fruit and is one of the fruit tree species traditionally used for planting along roads, streets or in parks in Poland. The berries are a valuable food source for many native bird species, which contribute to the spread of propagules over long distances by shedding seeds along with their faeces [36,37,38,39,40,41,42,43,44]. White mulberry is a kenophyte (synanthropic species of foreign origin, whether introduced after the 15th century) within the Polish flora [45]. It originated in the territory of present-day China and the first notes on its cultivation in Poland come from the 17th century. Local interest in this species considerably increased due to the development of silk production in Poland at the beginning of the 20th century, along with the establishment of the Experimental Silk Station in Milanówek in 1924. Numerous plantations have occurred since then, and the species has also become very popular in school yards and areas around workplaces. In addition, the 1950s saw the development of the Polish ‘Żółwińska wielkolistna’ variety [18,46,47]. 

There are numerous publications discussing the technology of white mulberry cultivation in various climatic and geographical regions of Poland, but so far, there are no reliable studies on the distribution of this species in urbanized areas, nor on the methods of its propagation, including both deliberate and spontaneous spread through self-seeding [18,46,48]. In our view, it is important to investigate the above. Moreover, the introduction of foreign tree taxa into the local environment requires a thorough investigation of related risks, such as the possibility of uncontrolled spreading of the species and subsequent displacement of the native flora [1,49,50]. It is estimated that one in ten newly introduced species proceeds to propagate out of control, one in a hundred are naturalized, while one in a thousand species that have been artificially introduced into the local environment may acquire invasive features, i.e., have a negative impact on native species, as well as on the local economy, health and life of the local inhabitants [51,52]. Although it is usually herbaceous plants that are thought to be potentially invasive, tree species also have the ability to spread uncontrollably [53,54]. In Poland, numerous cases of uncontrolled spread of non-native tree species have been reported both in cities and in forest complexes, while invasive spread of white mulberry has been observed, among others, in some states within the United States, Canada and Hungary [1,45,53,55,56,57,58,59,60]. 

Collecting data on the population of alien species within individual regions and forecasting potential changes in this population’s size and structure, as well as its possible impact on other organisms is 1 of the basic measures to reduce biological invasions, 1 of the 6 priority objectives of the European Biodiversity Strategy (EU 2020 Biodiversity Strategy) and part of the Regulation (EU) 1143/2014 on invasive alien species (the IAS Regulation) entered into force on 1 January 2015 [61,62]. The white mulberry population in Wrocław is probably one of the most sizeable in Poland. The aim of our study was to determine the exact size and structure of this population in the city of Wrocław and to examine the relationship between this structure and the intensity of anthropopressure and thermal conditions, which are varied within the area of Wrocław due to the urban heat island [1,63].

## 2. Materials and Methods

**Figure 1:**
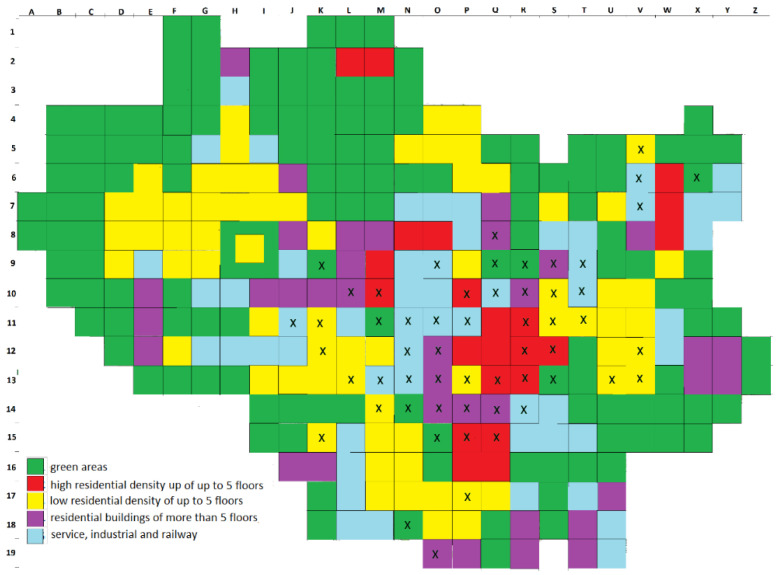
Environmental conditions. The type of development is generalized in a network of squares with an area of 1 square kilometer.

**Figure 2:**
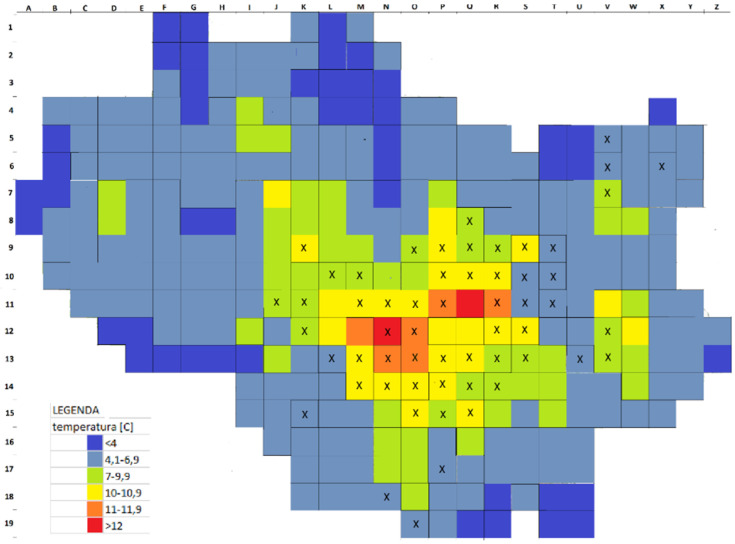
Thermal conditions, varied within the area of Wrocław due to the urban heat island generalized in a network of squares with an area of 1 square kilometre, in °C.

Our research spanned 2016–2019. The first step was to make an inventory of all white mulberry stands within the administrative boundaries of the city of Wrocław. Description of sites includes the location (address), along with the form of the individual or the type of planting (tree, hedge, a dense row, which density makes it impossible to distinguish specific individuals, and the position of the occurrence of juvenile seedlings, regardless of their number), scratched trunk or individuals with of trunk circumference greater than 10 cm as measured at the base (smaller circumference value means that the individual is considered a juvenile). The trees have also been divided into deliberate plantings and those propagated by self-seeding, i.e., growing in unusual places, such as in close proximity of walls or fences, in wastelands, rubble or railway areas. We used CBH (circumference at breast height) measurement based on the trunk circumference measured 130 cm above the ground, or in low-branched trees with several trunks, based on the sum of their circumferences, in order to distinguish 5 tree-size classes: (1) trees with CBH of less than or equal to 50 cm; (2) CBH of 51–100 cm; (3) CBH of 101–150 cm; (4) CBH of 151–200 cm; (5) CBH over 200 cm. For hedges and lanes, the total length of planting was measured. Mapping was carried out using the raster scan technique, involving basic research fields defined as squares of 1000 m × 1000 m, the total range of which covered the entire city area (Figure 1). For each 1-square-kilometre field, the total number of stands was determined, followed by the number of trees, the total length of hedges, the total length of rows and the number of self-seeding stands. The prevalence of mulberry in the basic research field was then determined. Site descriptions also included data on land use and type of buildings, illustrated in Figure 1, as well as thermal conditions, which are varied within the area of Wrocław due to the urban heat island (UHI) (Figure 2). The distribution of the urban thermal island in Wrocław is generalised in a grid of 1 km^2^ quadrants which has been prepared on the basis of meteorological materials gathered by the Department of Meteorology and Climatology of Wrocław University [63,64,65,66].

### Statistical Analysis

Statistical analyses were conducted using Statistica v 13 [67]. In order to determine the differences in mean values of natural and artificial re-occurrence of white mulberry (*Morus alba*) with regard to urbanization and thermal factors, variance analysis was conducted with relevance test between Tukey’s and Kruskal-Wallis tests. Principal component analysis (PCA) was conducted to determine the relationship between the variables.

In the statistical analyses, normal distribution was tested using Shapiro-Wilk W test, whereas the assessment for equality of variances–with Levene’s test. In case of lack of normality or equality of variances non-parametric analyses were conducted using the Kruskal-Wallis test. For other variables, the significance of differences was tested using one-way ANOVA with post-hoc testing of significance of differences using Tukey’s test. Principal component analysis (PCA) was conducted using the correlation matrix. The interpretation was conducted using the Kaiser criterion [68], taking into account exclusively eigenvalues greater than 1.00.

## 3. Results

### 3.1. White Mulberry Population Structure

**Figure 3:**
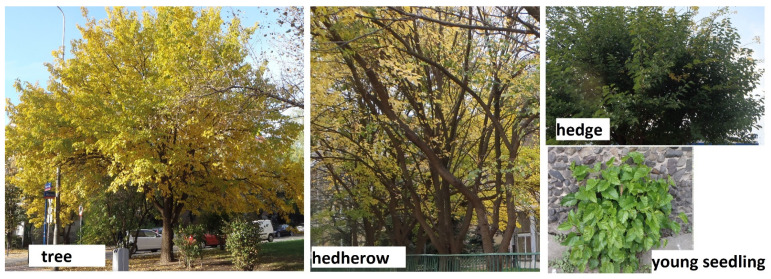
Mulberry growth types.

The research results revealed the occurrence of 1507 specimens of white mulberry in Wrocław agglomeration. The most numerous were trees (1366), which constituted 90.6% of the population. Hedges were less numerous, amounting to 2.7% of the population (40 specimens); adult seedlings–1.9% (28 specimens); young seedlings–4% (61 specimens); and hedgerows–0.8% (12 rows in the given square) (Figure 3).

**Figure 4:**
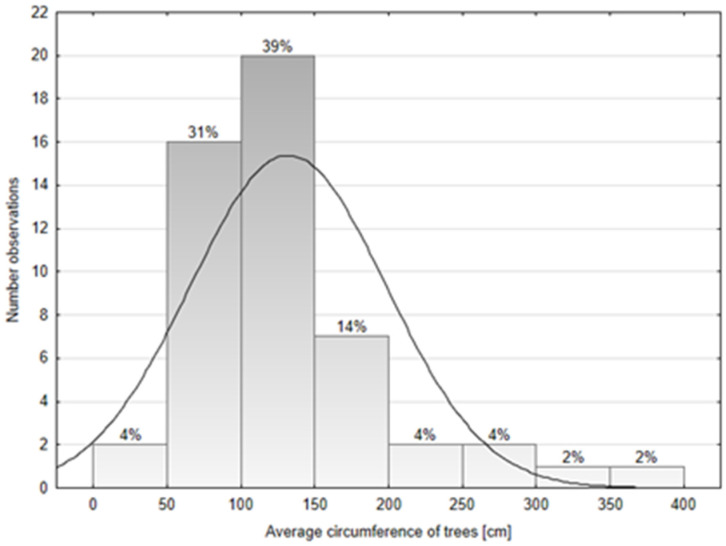
The distribution of *Morus alba* circumference. Percentage values represented the proportion of individuals trees of *Morus alba* in given circumference ranges (W Shapiro-Wilk test = 0.845; *p* ≤ 0.0001). The number of observations were calculated as mean for each square.

According to mean observations for each square, 20 trees had the circumference at breast height (CBH) between 100 and 150 cm, for 16 trees the diameter was between 50 and 100 cm, and 7 trees–between 150 and 200 cm (Figure 4).

### 3.2. The Influence of Urbanization on the Natural and Artificial Renewal of Morus Alba

The variance analysis did not show any differences in the mean values of natural or artificial renewal of *Morus alba* in the 5 types of urban residential density areas (Table 1).

The results of the principal components analysis (PCA) showed that the first 2 axes explain, respectively, 28.76 and 25.59 of the total variance (Table 2). Using the eigenvalue-1 criterion (the Kaiser criterion, line 3 of Table 2), the analysis demonstrated 72.93% of the total variance.

**Figure 5:**
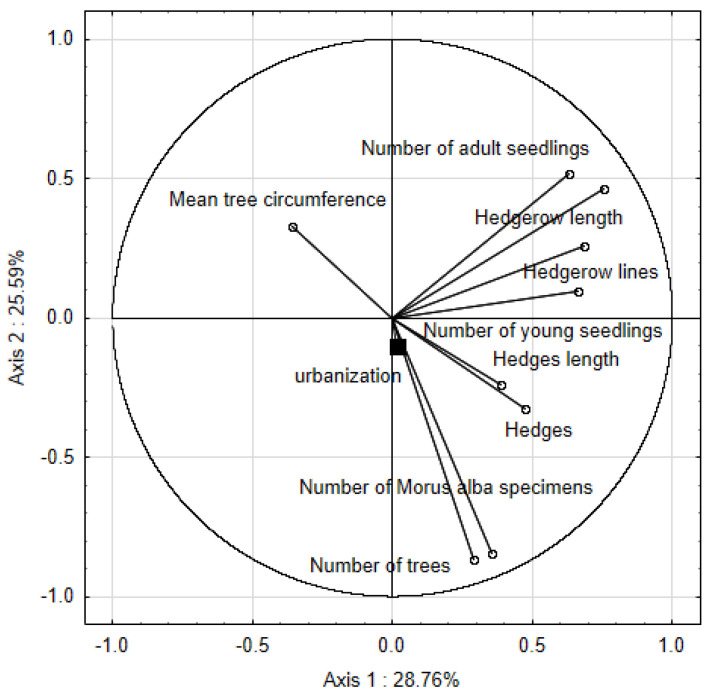
Principal component analysis conducted for parameters characterizing natural and artificial renewal of *Morus alba*. The level of urbanization was introduced into analyses as an additional variable.

In the PCA analysis (Figure 5) the second axis is correlated with the urbanization level (r = −0.98) and connected with the number of *Morus alba* specimens (r = −0.847), number of trees (r = −0.867), hedges (r = −0.328) and their length (r = −0.242).

### 3.3. The Influence of Thermal Factor on the Natural and Artificial Renewal of Morus Alba

Our calculations revealed a significant difference in the number of trees within different thermal categories defined for Wrocław. The highest quantity of *Morus alba* (H = 9.457; *p* = 0.024) and trees (H = 11.486; *p* = 0.009) was detected for thermal factor between 4.1 and 6.9 °C, whereas the lowest –in areas where the temperature is below 4 °C (Figure 2). For other variables that may affect both natural and artificial seeding no significant differences were found in mean values (Table 3).

In PCA results, the two first axes explain 27.08 and 24.68 percent of total variation (Table 4). Using the eigenvalue-1 criterion (the Kaiser criterion, line 4 of Table 1), the analysis demonstrated 80.63% of the total variance.

**Figure 6:**
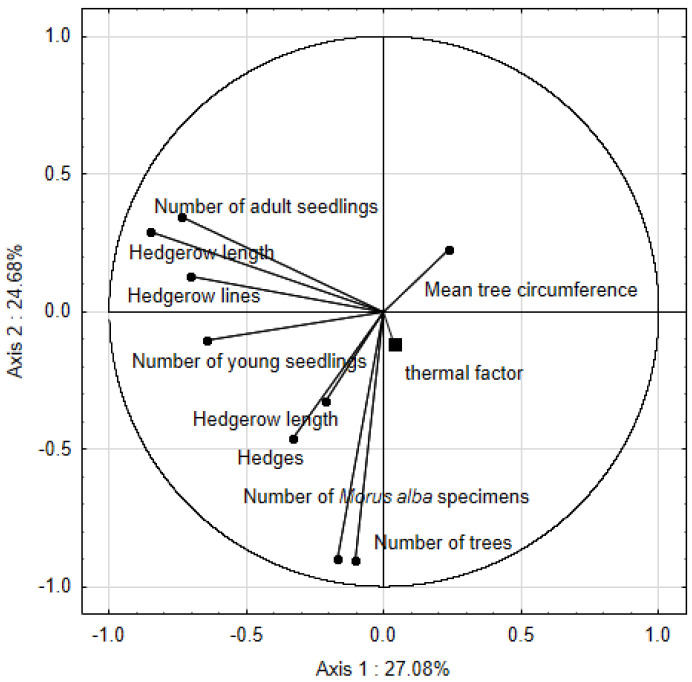
PCA conducted for parameters characterizing the natural and artificial renewal of *Morus alba*. The thermal factor was introduced into the analysis as an additional variable.

In the PCA analysis (Figure 6), the second axis is correlated with the thermal factor (r = −0.116), the number of trees (r = −0.903), the number of *Morus alba* specimens (r = −0.899), hedges (r = −0.460) and their length (r = −0.325).

## 4. Discussion

The allelopathic potential of white mulberry is relatively low [57,69]. A significant risk involved in the introduction of alien species is the possibility of the alien species crossing with representatives of native flora, threatening the purity of its genetic resources, and thus leading to their impoverishment. This phenomenon has been observed, among others in the European population of black poplar (*Populus nigra*), whose genetic diversity has clearly decreased as a result of hybridization with related, non-native taxa of poplars [70,71]. An analogous situation reported in Canada involved the formation of hybrids of white mulberry with the native red mulberry (*M. rubra*) [72]. It should be emphasized, however, that the Polish native flora includes no representatives of the genus Morus, or more broadly of the Moraceae family, which completely eliminates the possibility of the species in question having an impact on the gene pool of native taxa. However, its potential uncontrolled territorial expansion–as noted e.g., in Kentucky, US, where it is now one of the six most common non-native shrub species in the environment [55]–is still a cause for concern. One of the main causes of white mulberry infestation in both Iowa (US) and South Africa has been massive consumption of its fleshy, sweet fruit by local bird species, which results in its seeds being spread over long distances with bird droppings. It has also been demonstrated that the germination capacity of seeds that have passed through the bird’s digestive system is increased as compared to that of seeds obtained from whole, unused fruit [58,73]. Berries of this particular species are also popular with native bird species, such as sparrows, starlings and fieldfares, commonly found in Wrocław [74]. 

In Poland, white mulberry has been cultivated for over 300 years [45]. The existence of self-seeder, their perennial seedlings, and the formation of saplings in urban conditions are sometimes accepted as an indicator of the final stage of their synanthropization [75]. The observations made by Weber-Siwirska and Czekalski [2], related to generative renewal of trees and shrubs in Wrocław, indicated the presence of seedlings of 18 tree taxa, four of which were planted en masse, while no white mulberry seedlings were recorded. In the course of our research, spanning the years 2018–2019, the presence of juvenile seedlings of this species was established in 61 sites located within the city. Most had more than one seedling in a given site, while only 28 self-seeding adults were recorded. This is a negligible amount compared to the deliberate plantings, which constitute 94.1% of all detected positions. This demonstrates that it is possible to plant this species in Wrocław, but it is difficult for the seedlings to reach maturity, possibly due to tending or freezing, as is the case with other species originating from warmer climatic zones [76]. 

The dynamics of a tree population can be assessed based on its age structure. The individual age of mulberry is difficult to estimate using non-invasive methods, i.e., dendrometric measurements. Tree age-table used by Professor Longin Majdecki [77] to assess the age of trees based on the circumference of their trunk, does not provide data for this particular species. In the ordinance issued by the Polish Minister of Environment on fees payable for the removal of trees and shrubs mulberry is listed as a slow-growing tree due to the growth rate of its trunk, but it should be noted that in early stages of an individual’s development, the species is characterized by a high growth rate, following which, at the age of approximately 40–50 years, its growth dynamics clearly diminishes. The growth rate of the trunk and its thickness significantly depend on the growing conditions, showing great variation even within one species. We would like to stress that mulberry shows extremely high polymorphism. Moreover, it often branches low, thus creating multi-stemmed individuals [77,78,79,80,81,82,83,84,85,86,87,88,89,90,91]. Within the white mulberry population in Wrocław, the most numerous group consisted of trees that did not form part of dense rows (90.6% of all sites). Almost 2/5 of all the trees were characterized by the circumference of the trunk (or trunks) within the range of 100–150 cm, a slightly smaller group (31%) were trees with a circumference of 50–100 cm, while trees with a circumference of 150–200 cm accounted for 14%. It should be emphasized that the measured trunk circumference did not exceed 50 cm for only 1 in 25 trees. Observations by other authors, based on research conducted in six provinces of the West Pamir Valley (Tajikistan), where the species is a native taxon, indicate that in mixed populations consisting of both wild and cultivated individuals (orchards, backyard gardens) 64.5% on average are trees with a circumference at breast height of less than 1 metre, which roughly corresponds to the percentage of trees aged up to 50 years (60.8%) [91]. These observations may suggest that approximately 65% of all freely growing mulberry trees in Wrocław are over 50 years old. Research focusing on historical, more that century-old mulberry plantings cultivated for the purposes of silk production in the Goriška region in Slovenia, covering an area corresponding to half of the Wrocław area, showed a high diversity of plants, both in terms of their phenotypic and biochemical features, also linked to preference for specific forms and methods of cultivation, e.g. pruning techniques and types of plantings, as well as a clear dependence of their location on the average annual temperature [92]. Numerous studies conducted in urbanized areas have demonstrated that distribution of sites of other tree species may depend on the type of land use within a given area [93].

## 5. Conclusions

The analysis of the spatial structure of white mulberry population in Wrocław seems to confirm a significant correlation was found between the number of all sites in a given area, the number of trees, the number of hedgerow plantings, and the total length of hedges, and the level of urbanization, as well as the thermal factor. The highest frequency of mulberry occurrence, regardless of the type of planting, was recorded in squares consisting predominantly of service, industrial or railway areas, accounting for over 25% of all of the detected sites, with as many as 26% of trees growing not as part of dense rows. Plantings in the form of hedges were preferred in low residential density of up to 5 floors, with the largest number of such plantings in the low residential density areas, while the total length of hedges was the highest in the high residential density areas. However, it should be noted that the above relate to artificial plantings. It is surprising that there is no significant difference in the frequency of spontaneous renewals in areas with a different residential density structure and different annual average temperatures. This may indicate that the species does not manifest urbanophilic tendencies. The low number of seedlings reaching maturity is also a significant fact, leading to a conclusion that the taxon does not currently show signs of expansion in the city of Wrocław. However, the period between the introduction of a taxon and its excessive expansion can vary considerably, and in a temperate climate this process tends to be slower than in warmer climate zones. In Brandenburg, which is a region with a climate similar to that in Wrocław, tree species found to be invasive manifested this tendency after an average of 170 years, with twice as long needed in case of the walnut *Juglans regia* [53,54,94,95]. The invasive influence of trees and shrubs is difficult to assess on the basis of distribution alone [35,51,55,62,76,96,97]. Therefore, although white mulberry population in Wrocław does not seem to show the tendency for uncontrolled spontaneous spread, it is advisable to continue monitoring its current and future development. Our research has shown that the structure of the white mulberry population in Wrocław depends on the intensity of anthropopressure and thermal conditions. The above likely results from the deliberate planting of mulberry as a species resistant to urban conditions. The preferences of this species were of less importance in this case.

## Figures and Tables

**Table 1:** Mean values along with standard errors of parameters describing the natural (self-seeding) and artificial (intentional planting) of *Morus alba*. Different letters (a, b) indicate significant differences obtained after the Tukey’s test or Kruskal–Wallis test (*p* ≤ 0.05).

	Green Areas		High Residential Density up to 5 Floors		Low Residential Density of up to 5 Floors		Residential Buildings of More than 5 Floors		Service, Industrial and Railroad Areas	
	$\bar{x}$	SE	$\bar{x}$	SE	$\bar{x}$	SE	$\bar{x}$	SE	$\bar{x}$	**SE**
Number of *Morus alba* specimens	21.07 a	5.31	29.75 a	12.51	20.14 a	6.89	31.40 a	21.14	34.27 a	15.41
intentional planting										
Number of trees (excluding adult seedlings)	18.27 a	5.22	27.25 a	12.24	18.00 a	6.66	27.60 a	21.69	32.27 a	15.34
Mean tree circumference [cm]	149.94 a	17.15	149.73 a	36.25	121.22 a	15.27	107.11 a	20.30	117.53 a	18.55
Hedges [number]	0.73 a	0.21	0.88 a	0.52	1.00 a	0.36	0.40 a	0.24	0.40 a	0.16
Hedges length [m]	20.93 a	10.34	49.50 a	32.83	27.89 a	14.51	11.60 a	11.60	43.93 a	27.81
Hedgerow lines	0.20 a	0.14	0.50 a	0.19	0.14 a	0.10	0.60 a	0.40	0.00 a	0.00
Hedgerow length [m]	4.40 a	3.01	39.13 a	21.86	3.43 a	2.65	142.40 a	139.42	0.00 a	0.00
self-seeding										
Number of adult seedlings	0.53 a	0.29	0.25 a	0.25	0.29 a	0.29	1.60 a	1.60	0.40 a	0.19
Number of young seedlings	1.33 a	0.30	0.88 a	0.40	0.71 a	0.27	1.20 a	0.97	1.20 a	0.46

**Table 2:** The results of PCA main components analysis conducted for an additional variable representing urbanization level.

Value Number	Eigenvalue	% of Total Variance	Accumulated Eigenvalues	Accumulated of Total Variance [%]
1	2.59	28.76	2.59	28.76
2	2.30	25.59	4.89	54.34
3	1.67	18.59	6.56	72.93
4	0.80	8.85	7.36	81.78
5	0.76	8.39	8.12	90.18
6	0.45	5.04	8.57	95.21
7	0.26	2.87	8.83	98.08
8	0.17	1.92	9.00	100.00

**Table 3:** Mean values along with standard errors of parameters describing natural (self-seeding) and artificial (intentional planting) renewal of *Morus alba* with regard to thermal variable. Different letters (a, b, ab) indicate significant differences as showed by the Tukey’s test or Kruskal–Wallis test (*p* ≤ 0.05).

Class	<4		4.1–6.9		7–9.9		10–10.9	
	$\bar{x}$	SE	$\bar{x}$	SE	$\bar{x}$	SE	$\bar{x}$	SE
Number of *Morus alba* specimens	8.57 b	4.09	48.76 a	14.47	20.25 ab	4.17	30.20 ab	12.83
Intentional planting								
Number of trees (excluding adult seedlings)	6.14 b	3.88	46.47 a	14.49	18.05 ab	4.01	25.80 ab	11.93
Mean tree circumference [cm]	85.35 a	15.66	106.48 a	15.57	146.55 a	18.05	151.65 a	34.32
Hedges [number]	0.57 a	0.2	0.53 a	0.26	0.85 a	0.26	1.00 a	0.45
Hedges length [m]	28.04 a	15.21	9.18 a	4.69	29.15 a	13.76	56.00 a	33.88
Hedgerow lines	0.29 a	0.16	0.24 a	0.14	0.10 a	0.07	0.40 a	0.24
Hedgerow length [m]	53.43 a	49.81	4.00 a	2.4	13.45 a	9.52	10.80 a	6.68
Self-seeding								
Number of adult seedlings	0.86 a	0.62	0.35 a	0.17	0.30 a	0.21	0.80 a	0.49
Number of young seedlings	0.71 a	0.4	1.18 a	0.32	0.95 a	0.27	2.20 a	0.86

**Table 4:** The results of PCA conducted for an additional variable–thermal factor.

	Eigenvalue	% of Total Variance	Accumulated Eigenvalues	Accumulated of Total Variance [%]
1	2.44	27.08	2.44	27.08
2	2.22	24.68	4.66	51.76
3	1.59	17.71	6.25	69.47
4	1.00	11.16	7.26	80.63
5	0.76	8.45	8.02	89.07
6	0.48	5.30	8.49	94.37
7	0.30	3.38	8.80	97.75
8	0.20	2.25	9.00	100.00

## Data Availability

Not applicable.
